# Abiotic reduction of Cr(VI) by humic acids derived from peat and lignite: kinetics and removal mechanism

**DOI:** 10.1007/s11356-018-3902-1

**Published:** 2018-12-18

**Authors:** Suha T. Aldmour, Ian T. Burke, Andrew W. Bray, Daniel L. Baker, Andrew B. Ross, Fiona L. Gill, Giannantonio Cibin, Michael E. Ries, Douglas I. Stewart

**Affiliations:** 1 School of Civil Engineering, University of Leeds, Leeds, LS2 9JT UK; 2 School of Earth and Environment, University of Leeds, Leeds, LS2 9JT UK; 3 School of Physics and Astronomy, University of Leeds, Leeds, LS2 9JT UK; 4 School of Chemical and Process Engineering, University of Leeds, Leeds, LS2 9JT UK; 5 Diamond Light Source Ltd, Harwell Science and Innovation Campus, Didcot, Oxfordshire OX11 0DE UK

**Keywords:** Humic substances, Humic acids, Chromium, Contaminated land, Groundwater

## Abstract

Hexavalent chromium contamination of groundwater is a worldwide problem caused by anthropogenic and natural processes. We report the rate of Cr(VI) removal by two humic acids (extracted from Miocene age lignite and younger peat soil) in aqueous suspensions across a pH range likely to be encountered in terrestrial environments. Cr(VI) was reduced to Cr(III) in a first-order reaction with respect Cr(VI) concentration, but exhibited a partial order (~ 0.5) with respect to [H+]. This reaction was more rapid with the peat humic acid, where Cr(VI) reduction was observed at all pH values investigated (3.7 ≤ pH ≤ 10.5). ^13^C NMR and pyrolysis GC-MS spectroscopy indicate that the reaction results in loss of substituted phenolic moieties and hydroxyl groups from the humic acids. X-ray absorption spectroscopy indicated that at all pH values the resulting Cr(III) was associated with the partially degraded humic acid in an inner-sphere adsorption complex. The reaction mechanism is likely to be controlled by ester formation between Cr(VI) and phenolic/hydroxyl moieties, as this initial step is rapid in acidic systems but far less favourable in alkaline conditions. Our findings highlight the potential of humic acid to reduce and remove Cr(VI) from solution in a range of environmental conditions.

**Electronic supplementary material:**

The online version of this article (10.1007/s11356-018-3902-1) contains supplementary material, which is available to authorized users.

## Introduction

Chromium is a strategically important metal that is produced commercially from the chromite ore as sodium dichromate and similar chemicals (Wilbur et al. 2000; Jacobs and Testa 2005; Kogel et al. 2006). It is widely used in alloys, electroplating, leather tanning, timber treatment, wax, chromate pigments, refractories, ceramics, catalysts and organic acids (Barnhart 1997; Darrie 2001; Jacobs and Testa 2005; International Chromium Development Association 2016). Chromium has two environmentally stable oxidation states: Cr(III) and Cr(VI) (Pourbaix 1966; Brito et al. 1997). Cr(III) is an essential trace element for living organisms that has an influence on the biological function of the humans and animals (Lukaski 1999) and has a role in lipid, carbohydrate and glucose metabolism (Vincent 2000; Cefalu and Hu 2004). In contrast, Cr(VI) is toxic to plants (Chandra and Kulshreshtha 2004; Shanker et al. 2005), animals and humans (Costa 1997) and is classified as a mutagenic and carcinogenic material (Leonard and Lauwerys 1980; Kondo et al. 2003; Holmes et al. 2008).

Occasionally the manufacture and industrial use of chromium can lead to contamination of groundwater and soils (Burke et al. 1991; Puls et al. 1999; Geelhoed et al. 2002; Whittleston et al. 2011; Ding et al. 2016; Izbicki and Groover 2016; Matern et al. 2016). Natural processes have also lead to elevated chromium concentrations in groundwater above the World Health Organisation maximum for drinking water (50 μg/L; (WHO 2003)) at numerous locations around the world (Robertson 1991; Fantoni et al. 2002; Ball and Izbicki 2004; Steinpress 2005). For example, ultramafic rocks can have high Cr contents (Stueber and Goles 1967; Schwertmann and Latham 1986; Becquer et al. 2003), which is mainly in Cr(III) in the parent minerals, but can be oxidised to Cr(VI) during weathering, particularly by manganese (IV) oxides (Bartlett and James 1979; Eary and Rai 1987; Fendorf and Zasoski 1992). While Cr(VI) release can result from many processes, some of the most intractable environmental problems are associated with poor disposal of chromite ore processing residue (COPR) from the high-lime process. While this is an obsolete method for producing chromate chemicals, it is only now being phased-out in newly industrialised countries (e.g. India, China and Bangladesh; Darrie 2001; Gao and Xia 2011; Matern et al. 2016). As a result, there are numerous problematic legacy sites from this technology around the world (Higgins et al. 1998; Geelhoed et al. 2002; Stewart et al. 2007; Whittleston et al. 2011; Matern et al. 2017; Zhou et al. 2018). Water in contact with high-lime COPR has a pH > 12 and can have an aqueous Cr(VI) concentrations in excess of 1 mM (Higgins et al. 1998; Stewart et al. 2010; Matern et al. 2017). When such water inevitably escapes from abandoned waste piles into the geosphere, it produces Cr(VI) plume where the pH varies from hyperalkaline values close to source towards the natural soil value in the far field.

Due to its toxicity and potential mobility (as soluble anionic aqueous species such as CrO_4_^2−^, HCrO_4_^−^ and Cr_2_O_7_^2−^; Pourbaix 1966; Brito et al. 1997), the accidental release of Cr(VI) into terrestrial ecosystems is a significant cause for concern. In oxidising environments, surface complexation reactions with iron and aluminium oxide minerals can remove Cr(VI) from solution at acidic pH (Rai et al. 1989); however, at neutral and alkaline pH, adsorption to soil minerals is generally weak due to the presence of net negative surface charge at mineral surfaces (Rai et al. 1989; Langmuir 1997). Cr is far less mobile in reducing soil environments because aqueous Fe(II), Fe(II)-containing minerals and reduced sulphur compounds can rapidly reduce Cr(VI) to Cr(III) (Eary and Rai 1988; Rai et al. 1989; Eary and Rai 1991; Palmer and Wittbrodt 1991). Once reduced, Cr(III) will precipitate as Cr(OH)_3_ in circumneutral conditions (Pourbaix 1966) or, when reduced by Fe(II), as (Cr_x_ Fe_1−*x*_)(OH)_3_ (Sass and Rai 1987; Eary and Rai 1988; Rai et al. 1989).

Most soils contain organic matter, which plays an important role in the cycling of many elements in the environment (Gustafsson et al. 2001). Humic substances (the majority of soil organic matter; International Humic Substances Society 2007) are the dark-coloured, heterogeneous organic compounds produced by the decay and transformation of plant and animal residues by bacteria and fungi (Stevenson 1994; Swift 1999; Sutton and Sposito 2005; Brookes et al. 2008). The main humic precursor molecules are formed by depolymerisation and oxidation of plant biopolymers and proteins to produce molecules that contain unaltered polymer segments and phenolic, hydroxyl, carboxyl and amino residues (Wershaw 1986; Stevenson 1994; Swift 1999; Aro et al. 2005). However, there is still debate about how humic substances subsequently form. The traditional ‘polymer model’ assumes that the precursors are microbiologically synthesised into large randomly coiled polymeric macromolecules (Swift 1999), whereas recent evidence suggests that humic substances are supramolecular associations (Wershaw 1994; Kögel-Knabner 2000; Sutton and Sposito 2005). Amphiphilic precursor molecules cluster together into micelle-like particles (Wershaw 1999; Kögel-Knabner 2000), and other biomolecules from plant degradation become associated with either hydrophobic or hydrophilic domains (Piccolo et al. 1996; von Wandruszka 1998; Zang et al. 2000; Piccolo 2001; Simpson et al. 2002; Fan et al. 2004). While most evidence now supports this ‘micelle model’, it does not preclude development of polymer-type bonds, particularly as humic substances can potentially age and degrade over millions of years (Burdon 2001; Knicker et al. 2002; Sutton and Sposito 2005).

Cr(VI) can be reduced to Cr(III) by reaction with organic matter that contains phenolic, hydroxyl and aldehyde moieties (Lee and Stewart 1967; Wiberg and Schafer 1967; Elovitz and Fish 1995; Chen et al. 2015). The reaction with such moieties is thought to involve a chromate ester intermediate that can form with monomeric aqueous H_2_CrO_4_ and HCrO_4_^−^ species, with the redox step occurring during ester decomposition (Lee and Stewart 1967; Wiberg and Schafer 1967; Elovitz and Fish 1995). Reduction of Cr by this mechanism is rapid in acidic systems, but the rate decreases markedly with increasing pH (Lee and Stewart 1967; Elovitz and Fish 1995; Wittbrodt and Palmer 1997). Usually, it is assumed that Cr(VI) reduction by alcohol, phenolic and aldehyde moieties is negligible when ≥ pH 6 because chromate ester formation is less favourable with the CrO_4_^2−^ dianion, which is the dominant Cr(VI) species at high pH (Elovitz and Fish 1995). However, investigations of Cr(VI) mobility at sites contaminated with hyperalkaline (pH > 12) chromium ore processing residue leachate have observed Cr accumulation in organic-rich soils at ~ pH 10.5 (Higgins et al. 1998; Whittleston et al. 2011; Ding et al. 2016), indicating that high pH interactions may occur under field conditions and timescales not observed in short-term laboratory studies.

Although Cr(VI) reduction by humic substances has been well studied in acidic to neutral systems (Wittbrodt and Palmer 1995; Wittbrodt and Palmer 1997; Jardine et al. 1999; Huang et al. 2012), far less is known about potential reactions in the neutral to alkaline pH range relevant to COPR disposal sites. This knowledge gap is important because humic matter are one of the key soil component controlling Cr(VI) mobility in oxic near-surface environments. Further, the introduction of humic matter at a suitable pH point in a Cr(VI) plume could be the basis for groundwater treatment that mitigates environmental damage at otherwise intractable waste disposal sites. This study therefore investigates the reaction between aqueous Cr(VI) and humic acids derived from two sources (peat and lignin) over the range of pH values representative of an environment where an alkaline plume slowly buffers towards the natural pH value of the host soil. The objectives were to (i) investigate the rate at which Cr is removed from aqueous solution by the humic acids using batch exposure tests, (ii) to determine the oxidation state and local bonding environment of resulting solid-associated Cr using X-ray absorption spectroscopy (XAS) and (iii) identify changes humic acid functionality that resulted from the reaction using both ^13^C nuclear magnetic resonance (NMR) spectroscopy and pyrolysis-gas chromatography-mass spectrometry (PyGCMS). Together, these new data were used to develop a new understanding of the Cr(VI) reduction mechanism occurring with humic substances in the neutral to alkaline pH range.

## Materials and methods

### Humic acids

Humic acid is the humic fraction that is soluble at pH 12, but progressively precipitated as the pH is buffered to pH 2 (Stevenson 1994; Wershaw 1994; Sutton and Sposito 2005). Aldrich humic acid (AHA), a lignite derived humic acid (Poynton 2016), was acquired as a sodium salt (Sigma-Aldrich, UK). AHA used in some characterisation tests was further refined by dissolution and re-precipitation (rAHA). Peat humic acid (PHA) was obtained from Irish moss peat (Westland Horticulture Ltd., UK) by alkali extraction (Stevenson 1994). (See SI section S1.1 for full details.)

### Characterisation of humic acids

Characterisation analyses were carried out in triplicate. Ash contents were determined by ignition of moisture-free samples at 750 °C following ASTM D2974-07a (ASTM 2010). Percentages of C, H, N, S and O were determined using a Thermo Scientific FLASH 2000 CHNS/O elemental analyser (oxygen was determined in pyrolysis mode). The concentrations of inorganic constituents in AHA and PHA were determined by energy dispersive X-ray fluorescence (ED-XRF) spectroscopy (X-5000 Mobile XRF System—Olympus IMS). The total acidity and carboxylic acidity were measured using the barium hydroxide and Ca-acetate methods, respectively, and phenolic acidity was estimated by the difference (Schnitzer and Khan 1972). Carboxylic and phenolic acidity was also estimated through direct discontinuous base titrations that were conducted on 5 g/L humic acid suspensions in 0.5 N NaCl (see SI section 1.1) (Janoš et al. 2008).

### Cr(VI)-humic acid batch experiments

Humic acid powder (1 g) was added to DIW (90 mL) in 120-mL glass serum bottles (AHA was used as supplied whereas PHA came from the last step of the extraction protocol). Triplicate suspensions were equilibrated at pH values 3, 5, 7, 8.5, 9 and 11 using either 1 M HCl or NaOH. Samples were intermittently shaken, and the pH was adjusted until the pH value was stable for at least 1 day. The suspensions were then autoclaved at 121 °C for 15 min to ensure that the subsequent long-duration experiments were abiotic. After cooling, autoclaved DIW was added to each bottle to make up the volume to 96.7 mL, and the pH was readjusted, if necessary, to each target value. Air was flushed from the experiments by bubbling nitrogen through the suspensions. Finally, 3.3 mL of potassium chromate solution (1/30 M K_2_CrO_4_; Fluka, Germany) was added for a 100 mL final volume ([Cr(VI)] = 1100 μmol L^−1^). Bottles were sealed with butyl rubber stoppers with aluminium crimps (Sigma-Aldrich Company Ltd. UK). Control samples were prepared using the potassium chromate solution and autoclaved, N_2_ purged, DIW. Bottles were incubated in the dark at 20 ± 1 °C and periodically sampled aseptically for geochemical analysis. During sampling, bottles were shaken and 2 mL of suspension was extracted using N_2_ gas-filled syringes. Samples were divided for Cr(VI) and pH determination. Subsamples for Cr(VI) analyses were passed through a 3-kDa filter (Amicon ultra 0.5 centrifugal filter).

After testing (~ 50 days), further aqueous subsamples were taken for Cr(VI) and pH analysis, as described above, then an equal volume of aluminium sulphate solution (Al_2_(SO_4_)_3_.16H_2_O; 5 g/L) was added to the remaining sample to coagulate colloidal humic acid (HA) (two volumes were added to the pH 11 sample). The mixtures were shaken manually for a few seconds then centrifuged at 3226*g* for 1 min, and the supernatant was immediately separated. The solid phase was then washed three times with DIW and centrifuged. Half the solid sample was air-dried in an anaerobic cabinet and retained for XAS analysis. The other half was oven-dried at 100 °C. The supernatant and oven-dried solid samples were analysed for total Cr analysis as described below.

Similar AHA and PHA samples were prepared with an excess of Cr(VI) (8000 μmol Cr(VI)/g HA) to provide solid-phase samples for ^13^C NMR and PyGCMS analysis. The samples were prepared at pH 3 and allowed to equilibrate for 31 days before the solid phase was separated from the solution by filtration (control samples of AHA and PHA were equilibrated at pH 3).

Aqueous Cr(VI) concentrations were determined colourimetrically (method 7196A; USEPA 1992). Total Cr associated with the humic acid was determined after acid digestion (method 3050B; USEPA 1996). Total Cr in aqueous solutions and acid digestions were determined by inductively coupled plasma optical emission spectrometry (Thermo Scientific iCAP 7400 radial ICP-OES).

### X-ray absorption spectroscopy

Cr K-edge XAS spectra were collected from selected humic acid samples recovered from the low concentration Cr(VI) batch experiments (the ‘Cr(VI)-humic acid batch experiments’ section) on beamlines I18 and B18 at the Diamond Light Source, UK. Reference spectra were also collected for standard laboratory chemicals and precipitated Cr-hydroxide (Saraswat and Vajpe 1984). X-ray absorption near edge (XANES) spectra were summed and normalised using Athena v0.9.24 (Ravel and Newville 2005), and background subtracted extended X-ray absorption fine structure (EXAFS) spectra were fitted to model coordination environments using Artemis v0.9.24 (see SI sections S1.2 and S1.3 for details).

### Cross-polarisation magic-angle-spinning ^13^C-NMR spectroscopy

Humic acid samples were disaggregated and homogenised and packed into 4 mm diameter zirconium rotor tubes. Cross-polarisation magic-angle-spinning (CP/MAS) ^13^C-NMR spectra were obtained on a Bruker 400 MHz Avance II spectrometer, with a double-bearing magic-angle-spinning probe head (BL4 type) and a Bruker MAS II control unit (see SI for details). Chemical shifts were calibrated using an alpha-glycine spectrum (calibrated on the glycine peak at 43.5 ppm).

### Pyrolysis-gas chromatography-mass spectrometry

PyGCMS analysis was performed using a CDS 5000 series pyrolyser (CDS Analytical Inc., Oxford, PA, USA) connected to a Shimadzu QP2010 GC-MS (Shimadzu Corporation, Kyoto, Japan). Samples of approximately 2–3 mg of finely ground and homogenised humic acid were placed between quartz wool plugs in a quartz pyrolysis tube and pyrolysed at a heating rate of 20 °C per millisecond to 500 °C. The pyrolysates were initially trapped on a TENAX adsorbent trap before being desorbed into an Rtx 1701 capillary column (see SI section S1.5).

## Results

### Characterisation of humic acids

Aldrich humic acid produces 10× more ash upon ignition than PHA (27% and 2%). Refining AHA by alkali extraction/acid precipitation reduces the ash content to 18%. The principle inorganic elements in both humic acids are Al, Si, K, Fe and Ca (SI Table S2: Na is not detectable by ED-XRF), and these form ~ 18% of AHA by elemental mass, whereas these are about 0.5% of PHA by elemental mass. The detailed properties of AHA, rAHA and PHA are reported in full in the Supplementary Information (Table S1 and S2).

The C, H, N, S and O elemental compositions of rAHA and PHA are very similar. AHA contains proportionally more O than rAHA and PHA (assumed to be associated with the fraction removed by refining), but had a similar H/C ratio. The total acidity values of rAHA and PHA determined by the barium hydroxide method were 6.4 and 6.7 meq/g, respectively. The carboxylic acidities determined by the calcium acetate method were 3.1 and 2.6 meq/g, respectively, suggesting the phenolic acidities of rAHA and PHA were 3.3 and 4.1 meq/g, respectively. The carboxylic and phenolic acidities of rAHA and PHA determined from the base titrations were 3.7 and 3.3 meq/g (carboxylic) and 1.9 and 2.1 meq/g (phenolic), respectively (Table 1, SI Fig. S1).

**Table 1:** Carboxylic, phenolic and total acidity of the humic acids (meq/g)

Functional groups	rAHA	PHA
Total acidity—Ba(OH)_2_ method (1)	6.4 ± 0.4	6.7 ± 0.1
Carboxyl acidity—Ca-acetate method (2)	3.1 ± 0.1	2.6 ± 0.0
Phenolic acidity (difference between (1) and (2) above)	3.3	4.1
Carboxyl acidity—titration method (3)	3.7	3.3
Phenolic acidity—titration method (4)	1.9	2.1
Total acidity—titration method (sum of (3) and (4) above)	5.6	5.4

Note: Carboxylic and phenolic acidity were calculated from the base titrations following Ritchie and Purdue (2003)

### Aqueous Cr speciation and Cr(VI) removal rates determined after contact with humic acid

**Fig. 1:**
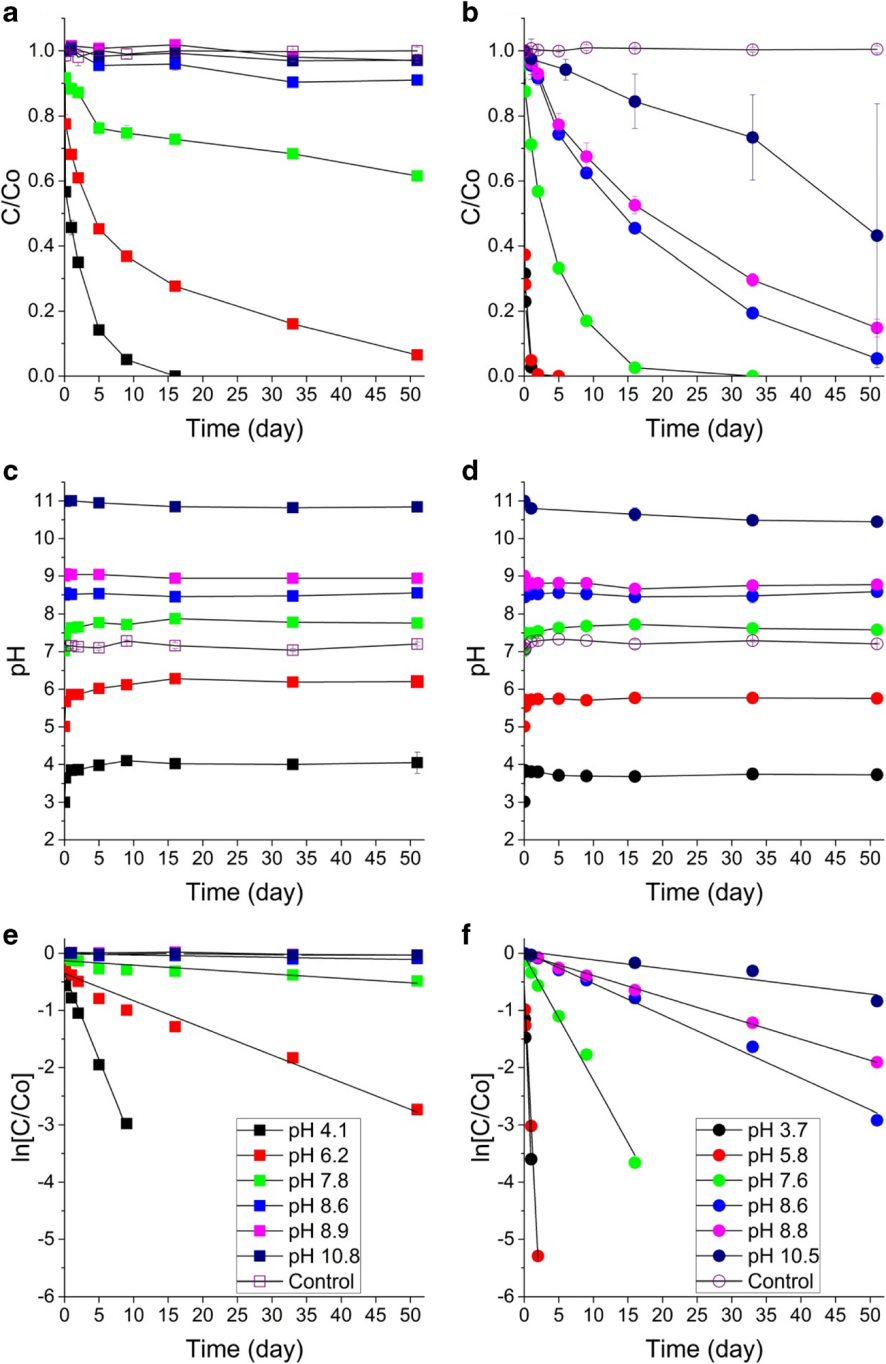
**a**, **b** Cr(VI) removal from free aqueous solution by AHA and PHA, respectively, at various initial pH values (C/C_0_ is the normalised Cr(VI) concentration; [Cr(VI)]_0_ = 1100 μM and [HA] = 1 g/100 mL); **c**, **d** solution pH of the AHA and PHA systems, respectively; **e**, **f** pseudo-first-order rate plots for the Cr(VI)-AHA and Cr(VI)-PHA reactions, respectively

The rate at which Cr(VI) was removed from free solution by AHA was dependent on the pH of the suspension (Fig. 1). The pH value of these systems changed slightly during the first 24 h, but quickly stabilised at the value used to name the systems. At pH 4.1 Cr(VI) was removed from solution over a period of about 15 days, whereas Cr(VI) removal at pH 6.2 took ~ 50 days. At pH 7.8 and pH 8.6, only partial Cr(VI) removal was observed after ~ 50 days (~ 40% and ~ 10% removal, respectively), with no detectable Cr(VI) removal above pH 9.

The rate at which Cr(VI) was removed from free solution by PHA was also dependent on the pH of the suspension. However, the reaction was significantly faster with PHA than with AHA (e.g. complete Cr(VI) removal at pH 5.8 took ~ 2 days), and the Cr(VI) removal was observed in all tests (85% and 55% of Cr(VI) were removed after ~ 50 days at pH 8.8 and pH 10.4, respectively).

**Fig. 2:**
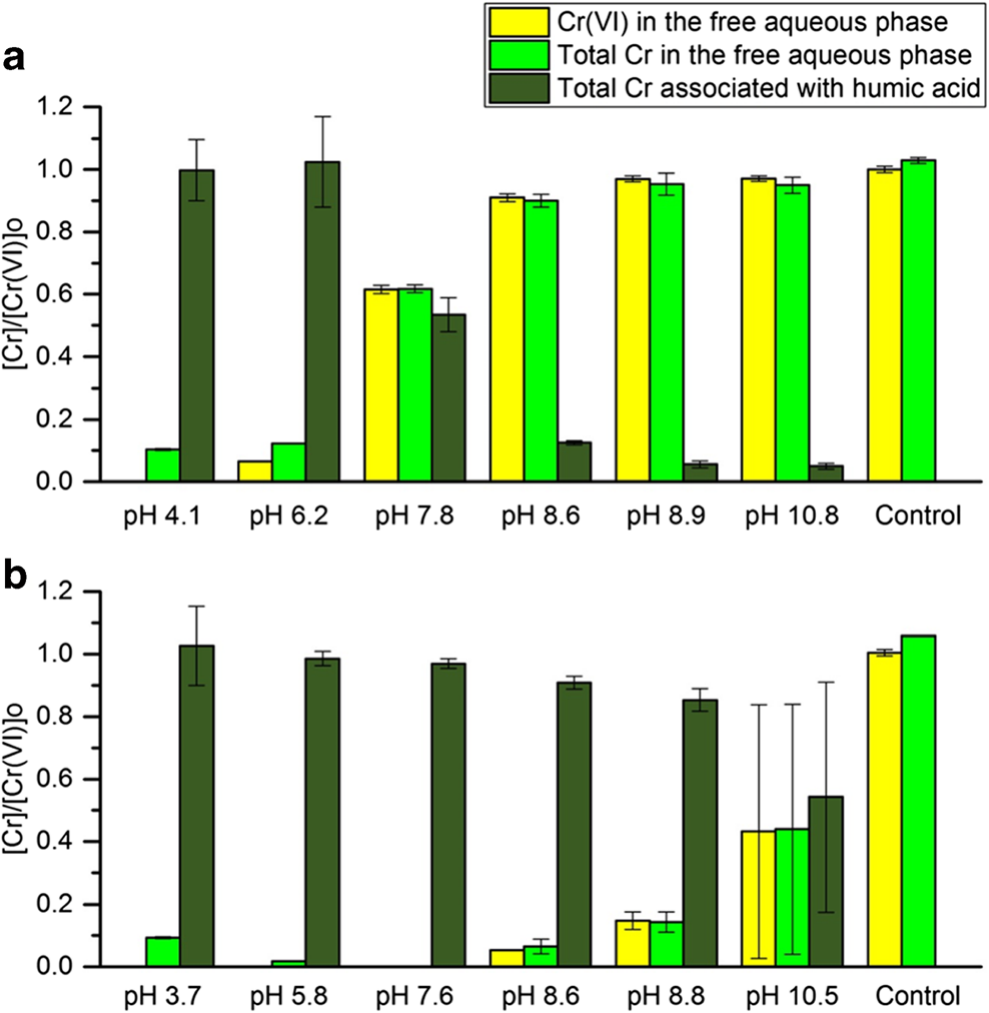
Speciation of **a** Cr(VI)-AHA and **b** Cr(VI)-PHA systems after 51 days. Solid to liquid ratio: 1 g/100 mL. Initial Cr(VI) concentration in the aqueous phase: [Cr(VI)]_0_ = 1100 μM

After 51 days, the partitioning of Cr between the free solution and the humic acid and the oxidation state of free aqueous Cr varied with the pH of the systems (Fig. 2). At pH 4.1 in the AHA system > 90% of the Cr(VI) initially in solution was transferred to the humic acid, and no free aqueous Cr(VI) was detected (the small amount of Cr remaining in free solution was attributed to aqueous Cr(III)). A similar pattern was observed at pH 6.2, although ~ 5% of the Cr remained as aqueous Cr(VI). At pH 7.8, about 60% of the Cr remained as aqueous Cr(VI). Above pH 8.5, there was very little Cr associated with the humic acid, and > 90% remained in the solution as Cr(VI).

In the presence of PHA, most of the Cr(VI) initially in free solution was transferred to the humic acid at pH < 8, and no free aqueous Cr(VI) was detected in these systems (although about 10% of the Cr remained in free solution at pH 3.7 presumably as Cr(III)). In the pH 8.6 and pH 8.8 PHA systems, most of the Cr was associated with the humic acid after 51 days, but ~ 5% and ~ 15% of Cr remained in solution as Cr(VI), respectively. However, at pH 10.5, there was more variation in the behaviour of the PHA system, so six replicates were tested. After 51 days, some Cr was associated with the humic acid in all replicates, but the amount of Cr(VI) remaining in free solution varied between 0 and 90% (average 45%).

AHA and PHA samples that were prepared with excess Cr(VI) for subsequent ^13^C NMR and PyGCMS analysis buffered the solution from pH 3 to ~ pH 7 in the long-term. AHA removed ~ 500 μmol Cr(VI)/g from solution. PHA removed ~ 1400 μmol Cr(VI)/g from solution.

### X-ray absorption spectroscopy

**Fig. 3:**
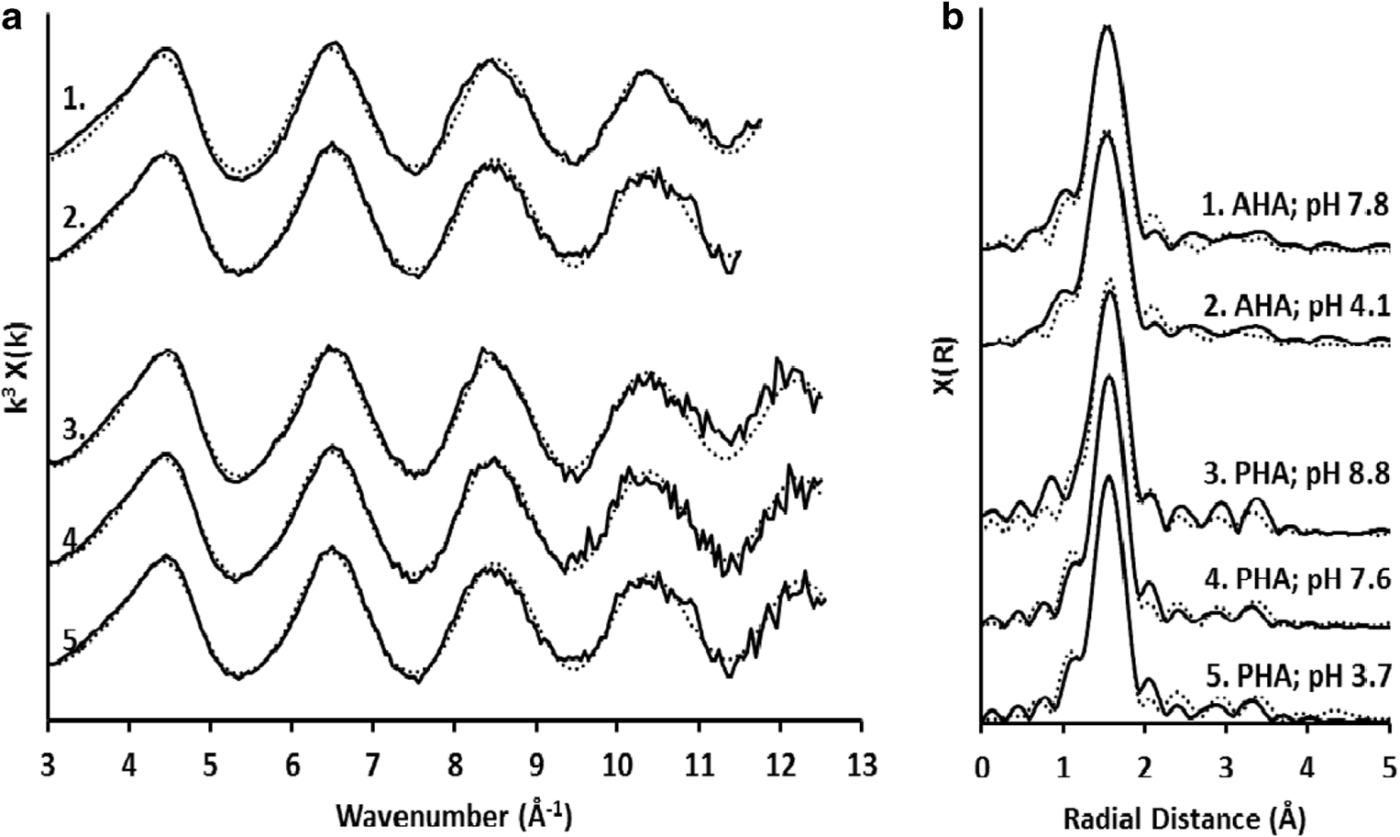
**a** Cr K-edge EXAFS data collected from Aldrich and peat humic acid samples, and **b** corresponding Fourier transformations. Dotted lines represent bit fit to data calculated in Artemis using pathways and parameters listed in SI Table S5

XANES spectra collected from both AHA and PHA samples that had been exposed to Cr(VI) lacked any evidence of the characteristic Cr(VI) pre-edge peak at 5994 eV (Peterson et al. 1996), indicating that only Cr(III) was present in solids regardless of the solution pH during the reaction (S.I. Fig. S2). The XANES spectra from both AHA and PHA sample were qualitatively similar and most closely resemble those collected from the Cr(III) aqueous or poorly crystalline hydrous Cr(OH)_3_ standards, lacking the detailed structure of the crystalline Cr_2_O_3_ standard (the absence of structure associated with Cr_2_O_3_ is probably indicative of Cr(III) binding with HA functional groups, since there is no Cr(III) observed in solution). EXAFS fitting revealed that all samples were best fit by single-scattering and multiple-scattering pathways associated with the Cr(III)O_6_ octahedra (i.e. 6 O atoms at 1.96–1.97 Å) and by the inclusion of additional Cr-C pathways between 2.91 and 3.00 Å (Fig. 3; SI Table S5). Attempts to fit the EXAFS spectra with additional Cr-Cr pathways at 3.0–3.1 Å produced final fits with unrealistically long Cr-Cr pathway lengths (3.3–3.9 Å) and the large Debye-Waller factors (0.009–0.010; indicative of overfitting) compared to other pathways and failed to improve the overall fit quality. Therefore, the data provided no evidence for Cr(OH)_3_ polymerisation that has been observed previously for some Cr(III)-humic acid associations (Gustafsson et al. 2014).

### Cross-polarisation magic-angle-spinning ^13^C NMR spectroscopy

**Fig. 4:**
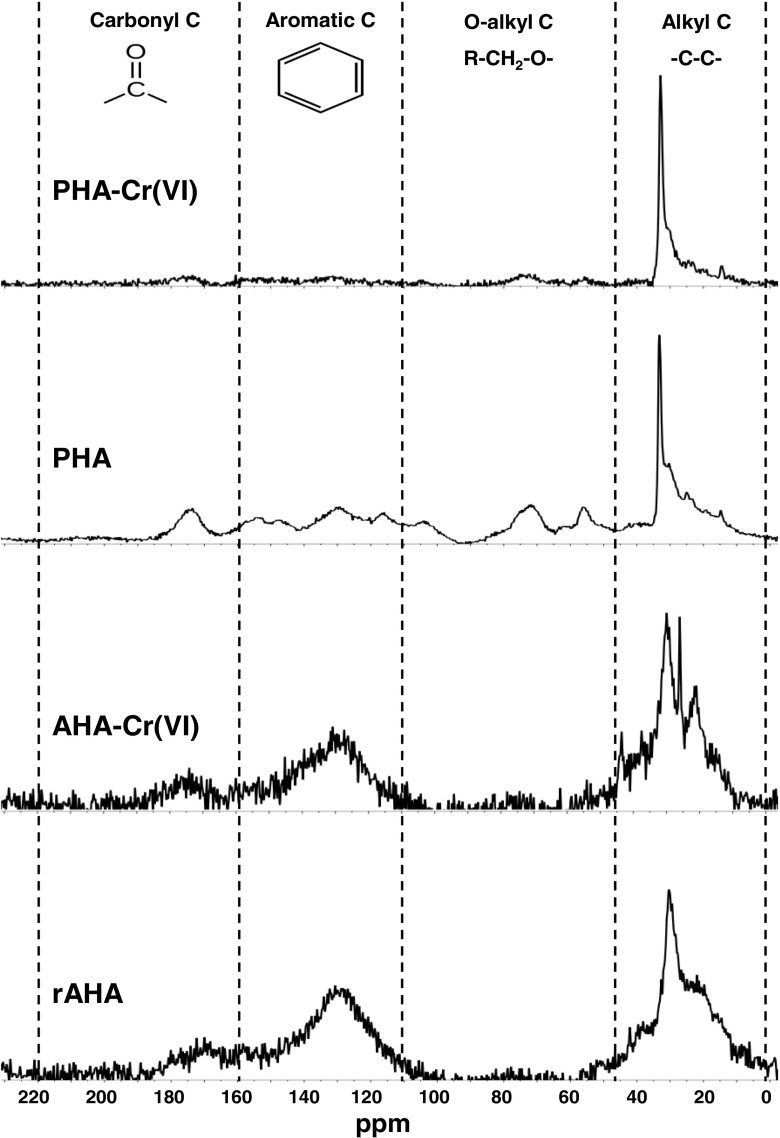
^13^C-NMR spectra of AHA and PHA before and after the reaction with excess Cr(VI) initially at pH 3. Curves are normalised to the equal area under the curves. The spectra are operationally divided into chemical shift regions characteristic of different C bonding environments (Golchin et al. 1997; Kögel-Knabner 2000)

Comparison of the ^13^C-NMR spectra of AHA and PHA indicates differences between the two materials (Table 2 and Fig. 4). Nearly 50% of the AHA spectrum is in the chemical shift range usually associated with alkyl C (0–45 ppm; carbon centres singly bonded to either C or H; Golchin et al. 1997; Kögel-Knabner 2000), with about 1/3 of the PHA spectrum occupying the same range. Conversely, ~ 25% of the PHA spectrum is in the range associated with alkyl C bonded singly to O (45–110 ppm), yet < 5% of the AHA spectrum is in this range. Approximately 40% of the AHA spectrum is in a range associated with alkene and aromatic C (110–160 ppm), whereas ~ 30% of the PHA spectrum is in this range. However, ~ 10% of both spectra are in the sub-range associated with aromatic C–O centres (140–160 ppm (Knicker et al. 2005)). Both humic acids have ~ 10% of their spectra associated with carbonyl C (160–220 ppm), and in both cases, this is mainly in the sub-range characteristic of carboxylic and ester moieties (160–185 ppm) (Knicker et al. 2005).

**Table 2:** Proportion of humic acid carbon in the different bonding environments before and after reaction with excess Cr(VI) determined by CP MAS ^13^C-NMR (spectra were operationally divided into characteristic chemical shift regions (Golchin et al. 1997; Kögel-Knabner 2000))

	AHA			PHA		
Type of organic carbon (% of total area)	Before reaction	After reaction	Difference	Before reaction	After reaction	Difference
Alkyl C (0–45 ppm)	47.1	52.0	+ 4.9	33.9	53.5	+ 19.6
O-alkyl C (45–110 ppm)	2.1	2.5	+ 0.4	24.8	14.7	− 10.0
Aromatic C (110–160 ppm)	38.7	33.9	− 4.8	27.8	17.7	− 10.0
Carbonyl C (160–220 ppm)	12.1	11.5	− 0.5	13.5	14.0	+ 0.5
Aromaticity* (%)	44.0	38.4	− 5.6	32.1	20.6	− 11.5

*Aromaticity is defined as (aromatic C)/(alkyl C + O-alkyl C + Aromatic C)

The ^13^C-NMR spectra of AHA and PHA both show changes due to the reaction with Cr(VI) in acidic solution (Table 2 and Fig. 4). The proportion of spectra usually associated with aromatic C has decreased by ~ 5% and ~ 10%, respectively. PHA also exhibits a ~ 10% decrease in the proportion of the spectrum associated with alkyl C singly bonded to O (from ~ 25 to ~ 15%), whereas AHA shows little change. In both cases, the proportion of the spectra usually associated with alkyl C (0–45 ppm) has increased by ~ 5% and ~ 20%, respectively. Neither material appears to exhibit any increase in the proportion of the spectrum associated with carbonyl C after reaction with Cr (160–220 ppm), but this may be the result of the shielding that occurs due to electron redistribution when carbonyl groups form complexes with Cr(III)(Zhang et al. 2017).

### Pyrolysis-gas chromatography-mass spectrometry

**Fig. 5:**
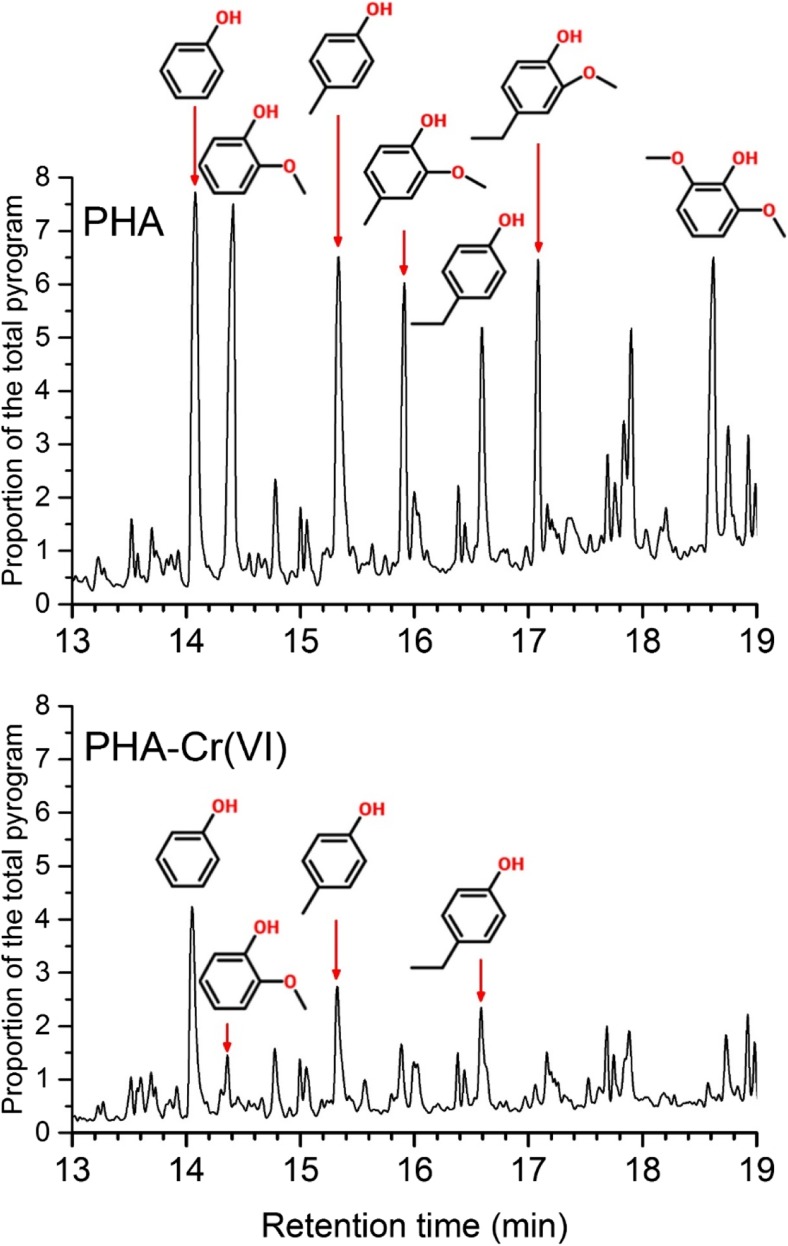
Partial pyrograms for PHA before and after reaction with excess Cr(VI) initially at pH3 showing a decrease in the relative size of spectral peaks associated with phenolic, methoxy-phenolic and other substituted phenolic fragments. Pyrograms have been scaled in proportion to the area that phenol represents of the total pyrogram. Full pyrograms and a table identifying the main thermal degradative products are presented in the Supporting Information

Direct quantitative comparison of functionality between the humic acid samples from PyGCMS is inappropriate due to the difference in detector response from different chemical fragments. However, examination of the pyrograms show differences before and after reaction with excess Cr(VI) in acidic solution and thus indicate the changes in humic acid functionality that resulted from the reaction. The reaction of PHA with excess Cr(VI) resulted in a large decrease in the relative size of peaks from products containing phenolic fragments and an increase in the relative size of peaks associated with long-chain aliphatic fragments (Fig. 5 and SI Fig. S3c, d). Peaks for methoxy-phenolic and other substituted phenolic compounds exhibited the largest decrease in relative size. The pyrograms for AHA were less well resolved (possibly a result of the higher ash content), but these also showed a decrease in the relative size of peaks associated with aromatic fragments (SI Fig. S3a, b).

## Discussion

### Relative differences in reactive sites in lignite and peat derived humic acids

Both the barium hydroxide and direct base titration methods indicate that rAHA and PHA have similar total acidity (per unit mass of the total material). However, the direct base titration and the difference between the barium hydroxide and calcium acetate methods indicate that PHA has 10–25% greater phenolic acidity than rAHA. More importantly, the ash remaining after a loss on ignition (18% in rAHA and 2% in PHA) is often a result of amorphous silica and aluminosilicate impurities present in the humic acid (Tan 1977). Such minerals can buffer pH during a titration producing ‘acidity’ in the phenolic range, and when allowance is made for the ash removed by refining (AHA is 27% ash), it is clear that the actual phenolic acidity of PHA is likely to be higher than that of AHA.

Chemical shift regions of the ^13^C NMR spectra indicate AHA and PHA contain similar proportions of carbonyl C centres associated with either carboxylic acid or ester groups (160–185 ppm). The ^13^C NMR spectra also indicate that the two HAs contain similar proportions of aromatic/alkene C centres in the range normally associated with aromatic C–O centres (140–160 ppm). ^13^C NMR cannot differentiate aromatic C bonded to O in phenolic moieties from those associated with an ether linkage (i.e. it cannot differentiate between Ph–O–H from Ph–O–C), but the differences in the phenolic acidity together with lower reactivity with Cr(VI) suggest that a larger proportion of the aromatic C–O centres in AHA may be associated with less reactive ether linkages (characteristic of phenolic polymers) than in PHA. AHA was extracted from Miocene age lignite (6–26 Ma; Germany), whereas PHA was extracted from Holocene age peat (< 12 ka, Ireland). As lignite is essentially compressed and heated peat, the differences in functionality of the two humic acids are probably associated with changes that occur during ageing of the parent materials. This is likely to have involved the formation of linking bonds between phenolic and other aromatic moieties (i.e. reactions similar to the polyphenol pathway assumed in the polymer model of humic substance formation; Stevenson 1994), as oxidative polymerisation of hydroxyphenols and toluenes can be catalysed by enzymes found in plants, fungi and bacteria (Martin and Haider 1980).

### Controls on the rate of Cr(VI) reduction with humic acid

XANES analysis indicates that the interaction of Cr(VI) with these humic acids resulted in a reduction to Cr(III) at all pH values tested. Also, the similarity of spectral details suggests that Cr(III) produced by reaction with humic acids resides in the same chemical environment regardless of the pH of the system. Changes in the ^13^C NMR spectra indicate that both humic acids suffered a loss of aromatic/alkene C during the reaction with Cr(VI) in acid solution. PHA also underwent a loss of O-alkyl C bonds (probably hydroxyl groups). Changes in the PyGCMS pyrograms confirm that both HAs suffered a loss of aromatic and an increase in aliphatic moieties during the reaction with Cr(VI) in acid solution. PyGCMS pyrograms suggest that the loss from PHA was principally of substituted phenolic aromatics, whereas with AHA, there was also a loss on non-phenolic aromatics. Collectively, these data suggest that the reduction of Cr(VI) to Cr(III) by humic acids involves a reaction with aromatic groups generally and phenolic moieties in particular. Changes in PHA suggest that aliphatic hydroxyl groups may also have reacted.

Humic acid samples for ^13^C NMR and pyrolysis GC-MS analysis were reacted with an excess of Cr(VI), and as a result, they buffered the pH from 3 to ~ 7 (indicating that H^+^ is consumed by the reaction between Cr(VI) and HA in acidic systems). These samples indicate that AHA can reduce ~ 500 μmol Cr(VI)/g and PHA ~ 1400 μmol Cr(VI)/g when the pH ≤ 7. Thus, in the longer term batch tests which were conducted with 110 μmol Cr(VI)/g, HA was available in excess when the pH ≤ 7. In this pH range, the rate of Cr(VI) removal by both HAs is first order with respect to the concentration of Cr(VI) species (Fig. 1e, f). The reaction may still be first order with respect to Cr(VI) concentration at pH > 7. At pH 7.8, AHA reduced only ~ 40 μmol/g Cr(VI) after 51 days, but the reaction exhibits an approximately linear relationship between log([Cr(VI)]) and time (*r*^2^ = 0.78; see SI Table S6). The reaction with PHA exhibited a linear relationship between log([Cr(VI)]) and time at pH 8.6 and 8.8 (*r*^2^ = 0.99 and 1.00, respectively).

The rates at which AHA and PHA reduced Cr(VI) is dependent on [H^+^], which decreases with the increasing pH. The rapidity of Cr(VI) removal from a solution made an accurate rate determination difficult at pH ≤ 4; however, least squares fitting of Eq. (1) to data where pH ≤ 9 yields the values of the exponent, *a*, of 0.48 for AHA and 0.40 for PHA (see SI Fig. S4).

1 $$ {k}_{\mathrm{obs}}={k}_0.{\left[{\mathrm{H}}^{+}\right]}^{\mathrm{a}} $$

This is consistent with the trend observed by Wittbrott and Palmer (Wittbrodt and Palmer 1995; Wittbrodt and Palmer 1997), who found that the rate of Cr(VI) reduction by soil fulvic acid and soil humic acid were both proportional to [H^+^]^0.45^ when pH ≤ 7.

### Mechanism of Cr(VI) reduction with humic acid

**Fig. 6:**
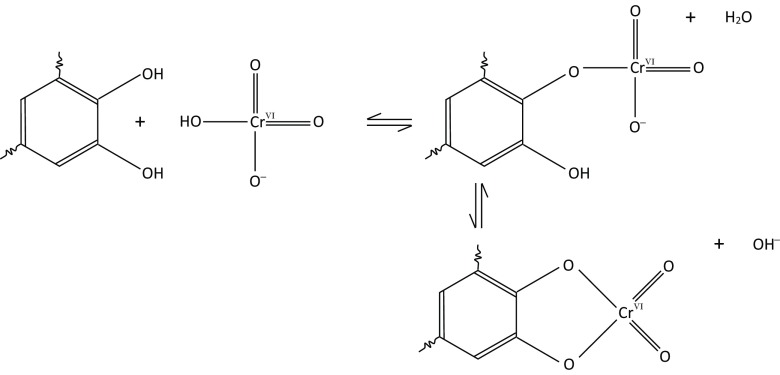
Formation of a chromate ester with phenolic moieties in humic acid, and a potential cyclic chromate ester with ortho-benzenediol moieties (Wiberg 1965)

Cr(VI) reduction by humic acid requires that Cr(VI) is first adsorbed in manner that facilitates electron transfer. Experimental studies using simple alcohols and phenolic compounds highlight the formation of a chromate ester as the first step in Cr(VI) reduction (Lee and Stewart 1967; Wiberg and Schafer 1967; Elovitz and Fish 1995). As alcohol and phenolic functional groups are common in humic acid, it is reasonable to expect similar interactions will occur in the experiments reported here, as illustrated in Fig. 6. Humic acids can contain vicinal diols (two hydroxyl groups attached to adjacent carbon atoms), like those found in the humic precursors caffeic, gallic and tannic acid (Nakayasu et al. 1999; Deiana et al. 2007), and these can also form cyclic chromate ester with Cr(VI) as illustrated in Fig. 6 (Wiberg 1965).

Chromate ester formation is rapid and reversible in acidic conditions (methylphenol reaches equilibrium in < 60 s at pH ≤ 5; Elovitz and Fish 1995). It occurs primarily with monomeric Cr(VI) species and is far more favourable with chromic acid and bichromate species than with the dianionic chromate species (Wiberg 1965; Lee and Stewart 1967; Wiberg and Schafer 1967; Elovitz and Fish 1995), probably because the Cr centre is more electrophylic in the monovalent bichromate than in the divalent chromate anion. Therefore, the rate at which Cr(VI) is reduced by alcohol and phenolic moieties declines sharply when pH ≥ 6 (Elovitz and Fish 1995). However, data presented here shows that Cr(VI) reduction proceeds slowly at alkaline pH, with the same eventual fate for the Cr(III) across the pH range (Fig. 3). This suggests that, while less favourable due to increased electrostatic repulsion, chromate ester formation occurs between deprotonated humic acids and the dianionic chromate species, with the result that Cr(VI) is reduced by humic acids at alkaline pH values on long timescales.

Reduction of Cr(VI) must have resulted in oxidation of the humic acids, but ^13^C NMR did not identify the functional groups produced. Oxidation of phenolic and alcohol groups by Cr(VI) usually produces ketones and carboxylic acids (Wiberg 1965; Rocek and Riehl 1967; Deiana et al. 2007), and thus, an increase in carbonyl range of the ^13^C NMR spectra would be anticipated. However, Zhang et al. (2017) showed that Cr(III) sorption to humic acids results in the formation of carbonyl-Cr(III) complexes, and shielding associated with electron redistribution causes a decrease in the ^13^C NMR signal from carbonyl groups. Thus, it is likely that Cr(VI) reduction by AHA and PHA resulted in the formation of additional carbonyl groups, but these were not detected by ^13^C NMR due to such shielding. This explanation is compatible with our Cr EXAFS data which suggests that the Cr(III) formed an inner-sphere adsorption complex with two C atoms.

The reduction of Cr(VI) by the humic acids was first order with respect to [Cr(VI)], so the rate-limiting step likely involves a single Cr-containing species. This step is probably chromate ester decomposition (Lee and Stewart 1967; Elovitz and Fish 1995; Wittbrodt and Palmer 1997), which can then result in transfer of one or two electrons to the chromate ion resulting in the production of either Cr(V) or Cr(IV) moieties (Wiberg 1965; Lee and Stewart 1967; Haight et al. 1971; Elovitz and Fish 1995). The reduction is also mixed order with respect to [H^+^] which is compatible with Cr(VI)-ester decomposition proceeding concurrently by multiple pathways (with 4-methylphenol ester decomposition can proceed concurrently by proton-activated pathways and a proton-independent pathway; Elovitz and Fish 1995), but it may also indicate that the equilibrium constant for the ester formation may be increasingly less favourable with H_2_CrO_4_, HCrO_4_^−^ and CrO_4_^2−^ species.

Metal reduction by vicinal diols can result in cleavage of the intervening C–C bond (Wiberg 1965; Deiana et al. 1992; Deiana et al. 1995; Deiana et al. 2007). Such ‘ring opening’ phenomena are consistent with the loss of aromatic and methoxy-phenolic groups observe by NMR and PyGCMS in this study. Cr(IV) moieties produced by electron transfer are unstable and will rapidly disproportionate to form Cr(III) and Cr(V), and Cr(V) can react with alcohol and phenolic groups via the chromate ester in much the same way as Cr(VI) (Wiberg 1965; Haight et al. 1971; Bruckner 2002). Therefore, through several cycles of absorption, ester formation, reduction and disproportionation steps, the Cr(VI) is likely to be eventually converted to the Cr(III) end product. Cr(III) formed from reduction of Cr(VI) remains associated with the partially degraded humic acid at all pH values, although with samples where pH < 4, small amounts of Cr(III) are also present in solution due to the protonation of humic acid surface sites which results in lower sorption of Cr(III) under acidic pH. The lack of Cr-Cr pathways in the Cr-humic acid inner-sphere adsorption complexes points to little or no aqueous Cr(III) accumulation after reduction, such that few Cr(III)-Cr(III) interactions occur during adsorption, and the formation of the Cr(III) dimers observed by Gustafsson et al. (2014) at high pH is not favoured.

### Implications

Cr(VI) is reduced to Cr(III) by reaction with humic substances over a wide range of pH values found in the environment. This reaction is rapid in acid and neutral conditions, and therefore, natural soil organic matter will reduce Cr(VI) transport through groundwater when it is present. Also, Cr(VI) contaminated groundwater could be treated by deploying humic substances within an engineered treatment scheme (such as a permeable reactive barrier). Reduction reduces Cr toxicity, and the resulting Cr(III) is strongly held by inner-sphere bonding with humic acids, which significantly reduces the opportunity for the spread of Cr(III) or reoxidation into mobile Cr(VI) species.

The rate at which Cr(VI) is reduced and the total capacity for Cr(VI) reduction are both proportional to the reactivity of the humic acid used and particularly the density of phenolic and hydroxyl sites in the humic substances. Thus, it is important to choose younger sources of organic matter (e.g. sewage sludge, compost), which contain more labile humic substances, to maximise treatment efficiency and longevity in real applications. This choice will be particularly important when pH > 7, where reaction rates are lower.

Treatment of Cr(VI) contaminated groundwater by permeable reactive barrier (PRB) is challenging in alkaline conditions as the reactive materials conventionally deployed within PRBs are not durable in this pH range (e.g. ZVI is passivated at high pH and Fe(II) containing substances, such as green rusts and ferric sulphate solutions, have very short active lifetimes). The reaction of Cr(VI) with humic acids will also not be easy to exploit within a conventional PRB, as it takes place on a timescale of weeks. However, a different remediation strategy may be appropriate, as humic acids become increasingly soluble as the pH rises and a large proportion of humic acids are mobile at high pH. Thus, humic acids will migrate with the groundwater until the pH is sufficiently buffered by reactions with soil minerals for precipitation, creating a diffuse reactive zone downstream of the intervention point.

## Electronic supplementary material

ESM 1 (DOCX 450 kb)
