# Supplementary material for: Abiotic reduction of Cr(VI) by humic acids derived from peat and lignite: kinetics and removal mechanism
Source: Environ Sci Pollut Res Int. 2018 Dec 18;26(5):4717–29. doi: 10.1007/s11356-018-3902-1 (PMC6394447; doi:10.1007/s11356-018-3902-1)
Supplement: Supplementary file 1 — (DOCX 450 kb) [file 11356_2018_3902_MOESM1_ESM.docx]

Supporting information

**Abiotic reduction of Cr(VI) by humic compounds representative of soil organic matter: kinetics and removal mechanism**

Suha T. Aldmour ^a^, Ian T. Burke ^b^, Andrew W. Bray ^b^, Daniel L. Baker ^c^, Andrew B. Ross ^d^. Fiona Gill ^b^, Giannantonio Cibin ^e^, Michael E. Ries ^c^, Douglas I. Stewart ^a,^*

^a^ School of Civil Engineering, University of Leeds, Leeds LS2 9JT, UK

^b^ School of Earth and Environment, University of Leeds, Leeds LS2 9JT, UK

^c^ School of Physics and Astronomy, University of Leeds, Leeds LS2 9JT, UK

^d^ School of Chemical and Process Engineering, University of Leeds, Leeds LS2 9JT, UK

^e^ Diamond Light Source Ltd, Harwell Science and Innovation Campus, Didcot, Oxfordshire, OX11 0DE, UK.

*Corresponding Author: [D.I.Stewart@leeds.ac.uk](mailto:D.I.Stewart@leeds.ac.uk),Tel.: +44 113 343 2287, School of Civil Engineering, University of Leeds, Leeds LS2 9JT, UK.

**Contents**

1. Additional experimental details
2. Supporting tables
3. Supporting figures

Number of pages: 19

Number of tables: 7

Number of figures: 4

**S1. Materials and methods**

*S1.1. Humic acid preparation and Standard chemical methods*

AHA was refined to produce rAHA. It was dissolved in DIW and the pH was increased to 12 using 0.5 N NaOH. The mixture was shaken for an hour, and acidified to pH <2 using 4 N HCl. The precipitate was collected by centrifugation (~3000 g, 45 min) and was washed twice with small amount of DIW and centrifuged. The final precipitate was dried at 40 ºC, and disaggregated using a mortar and pestle.

Peat (<2mm) was acid washed (0.1 M HCl) and then suspended in 0.5 N NaOH at a solid to liquid ratio of 1:10 and shaken for 24 hours. After centrifugation (~3000 g, 45 min) the supernatant was recovered and acidified to pH <2 by addition of 4 N HCl. The humic acid precipitate was recovered by centrifugation. This procedure was repeated twice more on the humic acid precipitate to maximise the removal of inorganic impurities. The precipitate was washed three times with small amount of DIW and centrifuged. The product was dried at 40 ºC and disaggregated using a mortar and pestle.

The absorbance ratio (E4/E6) of dilute humic acid solutions were determined at 465 and 665 nm using Thermo Scientific Biomate 3 spectrophotometer (Chen et al., 1977). The redox potential of 1:1 humic acid : DIW samples was measured at 24 ºC using Ultrameter II (Myron L Company).

Aqueous Cr(VI) concentrations were determined colorimetrically at 540 nm (Thermo Scientific Biomate 3 spectrophotometer) after reaction with 1,5- diphenylcarbazide under acidic conditions (USEPA, 1992). Total Cr associated with the solid phase was determined after acid digestion by following USEPA method 3050B (USEPA, 1996). The humic acid (0.15 - 0.25 g) was added to 5 ml of a 1:1 dilution of 70% HNO_3_ in DIW, and heated in a block heater (Grant QBD4) to 95 ± 5 ºC. A further 10 ml of 70% HNO_3_ was added gradually, followed by 1 ml DIW and 4 ml H_2_O_2_ (30%) was added gradually. When all effervescence had ceased and there was no further change in the general appearance of the sample, 3 ml of concentrated HCl was added. After cooling, the digestate solution was diluted to 25 ml with DIW, filtered using 0.2 µm PES syringe filter (Sartorius Minisart syringe filters), and then analysed for total Cr by ICP-OES. Total aqueous Cr was measured by Inductively Coupled Plasma Optical Emission Spectrometry (Thermo Scientific iCAP 7400 radial ICP-OES). The pH was measured using a pH meter (Hach HQ40d) calibrated daily at pH 4.01, 7, and 10.01. All of the reagents were of analytical grade. All glassware was washed with detergent, soaked overnight in 1 M HCl, and then rinsed with DIW three times.

Direct discontinuous base titrations were conducted where replicate experiments were equilibrated with different amounts of base (Janoš et al., 2008). Humic acid (0.25 g) was suspended aqueous in NaCl with predetermined volume of 0.5N NaOH in screw-cap bottles (total volume 50 mL, I ≈ 0.5 M). Samples bottles (100 ml polypropylene) were purged with nitrogen for several minutes, and sealed. Samples were equilibrated for 72 hours with intermittent shaking, and the final pH values were measured. Carboxylic and phenolic acidity were calculated from the base titrations following Ritchie and Purdue (Ritchie and Perdue, 2003).

*S1.2. X-ray absorption spectroscopy (XAS) data collection and analysis*

All samples and standards were analysed as pressed pellets (without diluent) sealed in Kapton tape in fluorescence mode (0.1 - 2 % Cr) or transmission mode (>2 % Cr) as appropriate. The Cr K-edge (5989 eV) XAS data presented in this study were gathered from two separate sessions, one on Beamline B18 (PHA samples) and one on Beamline I18 (AHA samples) at the Diamond Light Source.

At Beamline B18 the incident beam had a typical operating voltage of 3 GeV and a current of 300 mA. The x-rays at B18 are generated from a bending magnet source. The beam is vertically collimated by a Si mirror before passing through a double crystal Si monochromator. It was then focused onto the sample to give a spot size of 200 x 250 µm. Sr K edge (16105 keV) XAS spectra were gathered from fluorescence at room temperature x-rays using a 9 element Ge solid state detector. At this beamline multiple short scans (approx. 5 min.) were collected from each sample and each subsequent scan was assessed over time to ensure no X-ray induced changes in Cr speciation occurred.

At Beamline I18 the data was collected while operating at 3 GeV with a typical current of 250 mA, using a nitrogen cooled Si(111) double crystal monochromator and focussing optics. A pair of plane mirrors was used to reduce the harmonic content of the beam and Kirkpatrick-Baez mirrors were used to produce a relatively unfocused beam (approximately 50 µm diameter at the sample). Data was collected in fluorescence mode using a 9 element solid state Ge detector at room temperature. The sample stage was systematically moved between (~20 min.) scans to exposure a fresh part of the sample for each scan to reduce any possibility of X-ray induced changes in Cr speciation.

*S1.3 EXAFS data analysis and fitting*

Multiple XAS scans from each sample were summed and averaged using Athena v 0.8.056 (Ravel and Newville, 2005) to maximise the signal/noise ratio and XANES data was plotted. Cr K-edge EXAFS data was also collected and fit to molecular coordination models using Artemis v 0.8.056. Shell-by-shell fitting was performed by estimating initial parameters for single scattering pathways of backscattering atoms and then interactively refining these parameters. Specifically fits were determined by refining number of atoms (± 25 %), interatomic distances, Fermi energy and Debye-Waller factor (σ^2^). This procedure was performed initially with single scattering and multiple scattering pathways representing the CrO_6_ octahedral and subsequent Cr and/or C single scattering pathways were added. Additional pathways were then accepted only where they improved the overall fit quality by greater than 10 % (determined by reduction of the reduced Chi-squared measure of fit).

*S1.4. CP/MAS ^13^C-NMR*

HA samples that had been reacted with excess Cr(VI) (initially 8000 µmol/g at pH 3), and control HA samples that had been conditioned to pH 3, were disaggregated and homogenised and packed into a 4 mm diameter zirconium rotor tubes. Cross-polarisation magic-angle-spinning ^13^C-NMR spectra were obtained on a Bruker 400 MHz Avance II spectrometer, with a double-bearing magic-angle-spinning probe head (BL4 type) and a Bruker MAS II control unit. Measurement parameters were ~100.6 MHz frequency, 90 degree proton pulse length, 2.5 μs, 2 ms contact time, 5 s delay time, the spinning speed was 10 kHz. 5012 and 10,000 scans were collected and averaged for samples of HA and HA reacted with Cr(VI). Chemical shifts were calibrated using an alpha-glycine spectrum (calibrated on the glycine peak at 43.5 ppm).

Free induction decays (FIDs) obtained from ^13^C CP/MAS ^13^C-NMR experiments were Fourier transformed to obtain spectra and subsequently zero and first order phase corrections were applied. These NMR spectra were operationally divided into chemical shift regions; 0-45 ppm (alkyl C), 45-110 ppm (O-alkyl C),110-160 ppm (aromatic C) and 160-220 ppm (carbonyl C) (Golchin et al., 1997; Kögel-Knabner, 2000). These chemical shift regions are very similar to those used by Knicker et al. (Knicker et al., 2005), who further subdivided the aromatic region into aromatic-C bonded to C/H (110-140 ppm) and to O (140-160 ppm). For each spectrum the proportion of the area under the curve associated each chemical shift region was estimated by numerical integration (reported in Table 2 of the main paper).

*S1.5. Pyrolysis GC-MS*

HA samples were prepared by reaction with excess Cr(VI) (initially 8000 µmol/g at pH 3). Control HA samples were conditioned to pH3. Fine, homogenized HA samples (2-5 mg) were placed between quartz wool plugs in a small pyrolysis tubes. These were placed in the cold platinum filaments of the pyrolysis unit (CDS Pyroprobe® Model 5000). Samples were heated at a rate of 20 ˚C per millisecond to 500 °C (held for 20 seconds). The fragments (pyrolysates) were then carried by He gas into the gas chromatogram (GCMS-QP2010SE, Shimadzu). The pyrolysates were initially trapped on a TENAX adsorbent trap before being desorbed at 300 °C into the split/splitless injector of the GC inlet port via a heated transfer line with a split ratio of 20:1. Pyrolysates were separated on an Rtx-1701 capillary column (60 m long, 0.25 mm id, 0.25 μm film thickness) using a temperature programme of 40 °C (hold time 2 min), ramped to 280 °C at 6 °C/min (hold time 15 min) and a constant column head pressure of 2.07 bar. Pyrolysates were then transferred to the mass spectrometer, where they were detected by their mass to charge ratio using quadrupole mass detector. The absolute and relative intensity of each peak on the pyrogram was determined by performing the area integration using (Shimadzu GC solution) software.

**Table S1.** Properties of Aldrich and peat humic acid.

| Test | AHA | rAHA | PHA |
| --- | --- | --- | --- |
| Ash content % | 26.86±1.6 | 18.0±0.2 | 1.8±0.4 |
| C (%)^a^ | 51.1±0.2 | 59.9±2.8 | 60.1±1.4 |
| N (%) | 1.28±0.04 | 1.75±0.4 | 2.51±0.07 |
| H (%) | 4.73±1.05 | 5.26±1.56 | 5.57±0.29 |
| S (%)^b^ | 0 - 0.14 | 0-0.74 | 0-0.46 |
| O (%) | 42.8 ± 1.6 | 32.7±0.4 | 31.5±1.9 |
| H/C^c^ | 1.11 | 1.05 | 1.11 |
| O/C | 0.63 | 0.41 | 0.39 |
| (N+O)/C | 0.65 | 0.43 | 0.43 |
| E_4_/E_6_^d^ | 4.99±0.06 | 4.88±0.03 | 4.25±0.16 |
| Redox potential (mV) at 24 ºC ^e^ | - | 386 | 301 |

^a^ The elemental composition were calculated based on the moisture and ash free basis.

^b^ Some replicates were below the detection limit

^c^ H/C: atomic ratio of hydrogen to carbon. O/C: atomic ratio of oxygen to carbon. (N+O)/C: atomic ratio of sum of nitrogen and oxygen to carbon.

^d^ Ratio of absorbance at 465 and 665 nm determined following the method of Chen et al. (1977).

^e^ Redox potential of 1:1 humic acid:DIW samples measured at 24 ºC.

**Table S2.** Major inorganic impurities of AHA and PHA determined by XRF

| Element (W %) | AHA | PHA |
| --- | --- | --- |
| Mg | ND* | ND |
| Al | 4.14 ± 0.08 | 0.18 ± 0.02 |
| Si | 6.82 ± 0.04 | 0.24 ± 0.01 |
| S | 0.79 ± 0.00 | 0.70 ± 0.00 |
| K | 2.72 ± 0.01 | 0.05 ± 0.00 |
| Ca | 1.99 ± 0.01 | ND |
| Ti | 0.43 ± 0.00 | 0.00 ± 0.00 |
| Fe | 2.63 ±0.02 | 0.05 ± 0.00 |

*ND: not detected

**Table S3.** Data of Cr(VI) removal from aqueous solution by AHA over time as a function of pH, as depicted in Fig. 1 of the main text.

| **Time (day)** | **AHA pH 4.1** | | **AHA pH 6.2** | | **AHA pH 7.8** | | **AHA pH 8.6** | | **AHA pH 8.9** | | **AHA pH 10.8** | | **Control** | |
| --- | --- | --- | --- | --- | --- | --- | --- | --- | --- | --- | --- | --- | --- | --- |
|  | [Cr(VI)] µmol L^-1^ | pH | [Cr(VI)] µmol L^-1^ | pH | [Cr(VI)] µmol L^-1^ | pH | [Cr(VI)] µmol L^-1^ | pH | [Cr(VI)] µmol L^-1^ | pH | [Cr(VI)] µmol L^-1^ | pH | [Cr(VI)] µmol L^-1^ | pH |
| 0 | 1100 ± 0 | 3.0 ± 0.0 | 1100 ± 0 | 5.0± 0.0 | 1100 ± 0 | 7.0± 0.0 | 1100 ± 0 | 8.5± 0.0 | 1100 ± 0 | 9.0± 0.0 | 1100 ± 0 | 11.0± 0.0 | 1100 ± 0 | 7.2± 0.0 |
| 0.17 | 623 ± 6 | 3.7 ± 0.0 | 853 ± 32 | 5.7± 0.0 | 1008 ± 7 | 7.5± 0.2 | 1100 ± 11 | 8.5± 0.0 | 1100 ± 24 | 9.1± 0.0 | 1100 ± 22 | 11.0± 0.0 | 1083 ± 19 | 7.2± 0.0 |
| 1 | 502 ± 25 | 3.9 ± 0.0 | 750 ± 17 | 5.9± 0.0 | 972 ± 10 | 7.6± 0.1 | 1100 ± 22 | 8.5± 0.0 | 1122 ± 11 | 9.0± 0.0 | 1111 ± 0 | 11.0± 0.0 | 1111 ± 5 | 7.2± 0.1 |
| 2 | 385 ± 12 | 3.9 ± 0.0 | 671 ± 7 | 5.9± 0.0 | 960 ± 7 | 7.7± 0.0 | - | - | - | - | - | - | 1079 ± 29 | 7.1± 0.1 |
| 5 | 156 ± 11 | 4.0 ± 0.0 | 498 ± 17 | 6.0± 0.0 | 839 ± 22 | 7.8± 0.0 | 1045 ± 11 | 8.5± 0.0 | 1111 ± 14 | 9.0± 0.0 | 1078 ± 11 | 11.0± 0.0 | 1101 ± 3 | 7.1± 0.1 |
| 9 | 56 ± 4 | 4.1 ± 0.0 | 405 ± 11 | 6.1± 0.1 | 823 ± 25 | 7.7± 0.0 | - | - | - | - | - | - | 1024 ± 16 | 7.3± 0.0 |
| 16 | 0 | 4.0 ± 0.0 | 305 ± 1 | 6.3± 0.0 | 801 ± 8 | 7.9± 0.1 | 1056 ± 22 | 8.5± 0.0 | 1122 ± 6 | 8.9± 0.0 | 1089 ± 0 | 10.9± 0.0 | 1100 ± 6 | 7.2± 0.1 |
| 33 | 0 | 4.0 ± 0.0 | 177 ± 1 | 6.2± 0.1 | 752 ± 13 | 7.8± 0.0 | 990 ± 11 | 8.5± 0.1 | 1078 ± 14 | 8.9± 0.0 | 1067 ± 11 | 10.8± 0.1 | 1098 ± 6 | 7.0± 0.0 |
| 51 | 0 | 4.1 ± 0.3 | 72 ± 1 | 6.2± 0.0 | 677 ± 14 | 7.8± 0.0 | 1001 ± 11 | 8.6± 0.0 | 1067 ± 6 | 8.9± 0.0 | 1067 ± 11 | 10.8± 0.1 | 1100 ± 12 | 7.2± 0.1 |

**Table S4.** Data of Cr(VI) removal from aqueous solution by PHA over time as a function of pH, as depicted in Fig. 1 of the main text.

| **Time (day)** | **PHA pH 3.7** | | **PHA pH 5.8** | | **PHA pH 7.6** | | **PHA pH 8.6** | | **PHA pH 8.8** | | **PHA pH 10.5** | | **Control** | |
| --- | --- | --- | --- | --- | --- | --- | --- | --- | --- | --- | --- | --- | --- | --- |
|  | [Cr(VI)] µmol L^-1^ | pH | [Cr(VI)] µmol L^-1^ | pH | [Cr(VI)] µmol L^-1^ | pH | [Cr(VI)] µmol L^-1^ | pH | [Cr(VI)] µmol L^-1^ | pH | [Cr(VI)] µmol L^-1^ | pH | [Cr(VI)] µmol L^-1^ | pH |
| 0 | 1100 ± 0 | 3.0± 0.0 | 1100 ± 0 | 5.0± 0.0 | 1100 ± 0 | 7.0± 0.0 | 1100 ± 0 | 8.6± 0.0 | 1100 ± 0 | 9.0± 0.0 | 1100 ± 0 | 11.0± 0.0 | 1100 ± 0 | 7.2± 0.0 |
| 0.17 | 252 ± 4 | 3.8± 0.0 | 311 ± 17 | 5.5± 0.0 | 963 ± 14 | 7.5± 0.0 | 1091 ± 17 | 8.5± 0.0 | 1093 ± 13 | 8.8± 0.0 | - | - | 1099 ± 1 | 7.1± 0.0 |
| 1 | 30 ± 4 | 3.8± 0.0 | 54 ± 14 | 5.7± 0.0 | 783 ± 26 | 7.5± 0.0 | 1051± 31 | 8.5± 0.0 | 1056 ± 37 | 8.8± 0.1 | 1071 ± 68 | 10.8± 0.1 | 1108 ± 8 | 7.3± 0.0 |
| 2 | 0.0 | 3.8± 0.0 | 6 ± 2 | 5.7± 0.0 | 625 ± 20 | 7.5± 0.0 | 1007 ± 14 | 8.5± 0.0 | 1022 ± 40 | 8.8± 0.0 | - | - | 1102 ± 4 | 7.3± 0.0 |
| 5 | 0.0 | 3.7± 0.0 | 0.0 | 5.7± 0.0 | 366 ± 21 | 7.6± 0.0 | 818 ± 11 | 8.6± 0.0 | 851 ± 39 | 8.8± 0.1 | - | - | 1099 ± 3 | 7.3± 0.0 |
| 9 | 0.0 | 3.7± 0.0 | 0.0 | 5.7± 0.0 | 187 ± 13 | 7.7± 0.0 | 688 ± 11 | 8.5± 0.0 | 743 ± 46 | 8.8± 0.1 | - | - | 1111 ± 9 | 7.3± 0.0 |
| 16 | 0.0 | 3.7± 0.0 | 0.0 | 5.8± 0.0 | 29± 2 | 7.7± 0.0 | 501 ± 10 | 8.5± 0.0 | 578 ± 30 | 8.7± 0.1 | 930± 91 | 10.6± 0.2 | 1109 ± 6 | 7.2± 0.1 |
| 33 | 0.0 | 3.7± 0.0 | 0.0 | 5.8± 0.0 | 0.0 | 7.6± 0.0 | 213 ± 20 | 8.5± 0.2 | 326 ± 22 | 8.8± 0.1 | 807 ± 145 | 10.5± 0.1 | 1103 ± 8 | 7.3± 0.0 |
| 51 | 0.0 | 3.7± 0.0 | 0.0 | 5.8± 0.0 | 0.0 | 7.6± 0.0 | 59 ± 0.0 | 8.6± 0.0 | 163 ± 31 | 8.8± 0.0 | 475 ± 447 | 10.5± 0.1 | 1106 ± 11 | 7.2± 0.0 |

**Table S5.** Cr K-edge EXAFS fits, where N is the Occupancy, r is the interatomic distance, σ^2^ is the Debye–Waller Factor and reduced χ^2^ and *R* are the goodness of fit parameters. Initial set of goodness of fit parameters relate to fits including just CrO_6_ octahedral pathways; second set of parameters relates to best fits including an additional Cr-C pathway (data shown). Uncertainties in the last digit shown in parentheses. MS = multiple scattering pathways as indicated.

| **Experiment Description** | **Pathway** | **N** | **r (Å)** | **σ^2^ (Å^2^)** | **χ^2^; *R*** |
| --- | --- | --- | --- | --- | --- |
| **Aldrich Humic acid pH 4.1**  δe0 = -1(1) S^2^_0_ = 0.77(5) | Cr-O | 6 | 1.96(1) | 0.003(1) | 666; 0.027 |
|  | Cr-C | 2 | 2.96(3) | 0.008(4) | 434; 0.016 |
|  | MS CrO_6_ | 3 x 6 | 3.93(2) | 0.005(2) |  |
| **Aldrich Humic acid pH 7.8**  δe0 = 0(1) S^2^_0_ = 0.81(7) | Cr-O | 6 | 1.96(1) | 0.003(1) | 833; 0.026 |
|  | Cr-C | 2 | 2.91(5) | 0.007(7) | 643; 0.018 |
|  | MS CrO_6_ | 3 x 6 | 3.91(2) | 0.005(2) |  |
| **Peat Humic acid pH 3.7**  δe0 = 1(1) S^2^_0_ = 0.78(5) | Cr-O | 6 | 1.97(1) | 0.002(1) | 137; 0.017 |
|  | Cr-C | 2 | 2.98(4) | 0.007(4) | 93; 0.011 |
|  | MS CrO_6_ | 3 x 6 | 3.94(2) | 0.005(2) |  |
| **Peat Humic acid pH 7.6**  δe0 = 1(1) S^2^_0_ = 0.80(5) | Cr-O | 6 | 1.97(1) | 0.002(1) | 74; 0.014 |
|  | Cr-C | 2 | 3.00(5) | 0.008(4) | 55; 0.010 |
|  | MS CrO_6_ | 3 x 6 | 3.94(2) | 0.005(2) |  |
| **Peat Humic acid pH 8.8**  δe0 = 2(1) S^2^_0_ = 0.83(5) | Cr-O | 6 | 1.97(1) | 0.002(1) | 143; 0.017 |
|  | Cr-C | 2 | 3.00(5) | 0.009(9) | 117; 0.014 |
|  | MS CrO_6_ | 3 x 6 | 3.95(2) | 0.005(2) |  |

**Table S6.** Rate constants and half-lives for Cr(VI) reduction by AHA and PHA at various pH values.

|  | pH | K (hr^-1^) | R^2^ | Half life (hr) |
| --- | --- | --- | --- | --- |
| AHA | 4.1 | 1.3 × 10^-2^ | 0.97 | 55 |
|  | 6.2 | 2.0 ×10^-3^ | 0.96 | 350 |
|  | 7.8 | 3.0 ×10^-4^ | 0.78 | 2300 |
|  | 8.6 | 8.0 × 10^-5^ | 0.83 | 8700 |
| PHA | 3.7 | 1.3 × 10^-1^ | 0.91 | 5.4 |
|  | 5.8 | 1.0 ×10^-1^ | 0.97 | 6.9 |
|  | 7.6 | 9.1 × 10^-3^ | 0.99 | 76 |
|  | 8.6 | 2.3 ×10^-3^ | 0.99 | 300 |
|  | 8.8 | 1.5 × 10^-3^ | 1.00 | 460 |
|  | 10.5 | 6.0 ×10^-4^ | 0.917 | 1200 |

**Table S7.** Principal organic compounds in AHA and PHA before, and after reaction with 8000 µmol Cr(VI) at pH3, identified by PyGCMS (see SI Figure S3). Compounds separated in the pyrograms produced by Py-GC-MS are identified using a NIST Standard Reference database (NIST11s MS library). All compounds listed exhibited a good match (>90%) with the database.

| **Peak No.** | **Compound** | **Aromatic / Aliphatic** | **Relative Area×100%** | | | |
| --- | --- | --- | --- | --- | --- | --- |
|  |  |  | **rAHA** | **AHA-Cr(VI)** | **PHA** | **PHA-Cr(VI)** |
| 1 | Propylene oxide | Aliphatic | - | 0.19 | - | - |
| 2 | Furan, 2-methyl- | Aromatic | - | - | 1.68 | - |
| 3 | Benzene | Aromatic | 1.16 | 0.57 | - | - |
| 4 | Toluene | Aromatic | 2.19 | 0.62 | 3.52 | 2.55 |
| 5 | Pyridine | Aromatic | - | - | 1.44 | 1.00 |
| 6 | Pyridine, 2-methyl- | Aromatic | - | - | - | 0.56 |
| 7 | Ethylbenzene | Aromatic | 0.40 | - | - | - |
| 8 | Dimethyl benzene | Aromatic | 2.44 | 0.28 | - | - |
| 9 | Dimethyl benzene | Aromatic | 0.68 | - | - | - |
| 10 | 1,3,5,7-Cyclooctatetraene | Aliphatic | - | - | 0.62 | 0.46 |
| 11 | 1H-Pyrrole, 2-methyl | Aromatic | - | - | 0.48 | 0.46 |
| 12 | Anisole | Aromatic | - | - | - | 0.62 |
| 13 | 1H-Pyrrole, 2-methyl | Aromatic | - | - | 0.98 | 0.65 |
| 14 | Benzene, 1-ethyl-4-methyl | Aromatic | 0.52 | - | - | - |
| 15 | 2-Cyclopenten-1-one, 2-methyl- | Aliphatic | - | - | - | 0.50 |
| 16 | Trimethyl benzene | Aromatic | 0.77 | - | - | - |
| 17 | Trimethyl benzene | Aromatic | 1.56 | - | - | - |
| 18 | Benzene, 1-methoxy-2-methyl- | Aromatic | - | - | - | 0.50 |
| 19 | 2-Furancarboxaldehyde, 5-methyl- | Aromatic | - | - | 0.91 | - |
| 20 | 2-Cyclopenten-1-one, 3-methyl- | Aliphatic | - | - | - | 0.46 |
| 21 | 1-Dodecene | Aliphatic | - | - | - | 0.29 |
| 22 | Benzene, 1-ethyl-4-methoxy- | Aromatic | - | - | - | 0.33 |
| 23 | Acetophenone | Aromatic | - | - | - | 0.38 |
| 24 | Phenol | Aromatic | 5.09 | - | 7.72 | 4.24 |
| 25 | Phenol, 2-methoxy- | Aromatic | - | - | 6.88 | 0.64 |
| 26 | Phenol, 2-methyl- | Aromatic | 0.71 | - | 1.17 | 1.13 |
| 27 | Tridecane | Aliphatic | - | - | 0.57 | 0.60 |
| 28 | 1-Tridecene | Aliphatic | - | - | 0.56 | 0.78 |
| 29 | p-Cresol (4 methyl phenol) | Aromatic | 0.48 | - | 5.99 | 2.07 |
| 30 | Cresol (2-methoxy-4 methylphenol) | Aromatic | - | - | 3.71 | - |
| 31 | Phenol, 2,4-dimethyl | Aromatic | - | - | - | 1.23 |
| 32 | Tetradecane | Aliphatic | -- | - | 0.62 | 0.53 |
| 33 | 1-Tridecene | Aliphatic | - | - | 0.36 | 0.45 |
| 34 | Phenol, 4-ethyl- | Aromatic | - | - | 3.54 | 2.01 |
| 35 | Phenol, 4-ethyl-2-methoxy- | Aromatic | - | - | 3.77 | - |
| 36 | Tetradecane | Aliphatic | - | - | 0.75 | 0.67 |
| 37 | 1-pentadecene | Aliphatic | - | - | 0.67 | 0.55 |
| 38 | Phenol, 2,6-dimethoxy- | Aromatic | - | - | 4.72 | - |
| 39 | Naphthalene, 2,6-dimethyl- | Aromatic | 0.78 | - | - | - |
| 40 | Indole | Aromatic | - | - | 1.02 | 0.92 |
| 41 | Tetradecane | Aliphatic | - | - | 0.98 | 0.92 |
| 42 | 1-Pentadecene | Aliphatic | - | - | 0.44 | 0.56 |
| 43 | Trans-Isoeugenol | Aromatic | - | - | 0.61 | - |
| 44 | 1H-Indole, 3-methyl- | Aromatic | - | - | - | 0.72 |
| 45 | Octadecane | Aliphatic | 0.35 | - | 1.06 | 1.24 |
| 46 | 1-Pentadecene | Aliphatic | - | - | 0.62 | 0.62 |
| 47 | Naphthalene, 2,3,6-trimethyl- | Aromatic | 0.38 | - | - | - |
| 48 | Naphthalene, 2,3,6-trimethyl- | Aromatic | 0.31 | - | - | - |
| 49 | 1-Dodecanol, 3,7,11-trimethyl- | Aliphatic | 0.68 | - | - | - |
| 50 | 1-Dodecanol, 3,7,11-trimethyl- | Aliphatic | - | - | - | 2.03 |
| 51 | Naphthalene, 2,3,6-trimethyl- | Aromatic | 1.20 | - | - | - |
| 52 | Apocynin | Aromatic | - | - | 0.82 | - |
| 53 | Octadecane | Aliphatic | - | - | 0.90 | 1.36 |
| 54 | 1-Octadecene | Aliphatic | - | - | 0.6 | 1.16 |
| 55 | Nonadecane | Aliphatic | - | - | 1.32 | 2.05 |
| 56 | 1-Nonadecene | Aliphatic | - | - | 0.68 | 0.96 |
| 57 | 2-Pentadecanone, 6,10,14-trimethyl- | Aliphatic | - | - | - | 0.70 |
| 58 | Octadecane | Aliphatic | 0.34 | - | 0.89 | 1.53 |
| 59 | 1-Nonadecene | Aliphatic | - | - | 0.49 | 1.05 |
| 60 | 1-Nonadecene | Aliphatic | - | - | - | 0.46 |
| 61 | 2-Heptadecanone | Aliphatic | - | - | - | 0.32 |
| 62 | Ethanone, 1-(4-hydroxy-3,5-dimethoxyphenyl)- | Aromatic | - | - | 0.35 | - |
| 63 | Heneicosane | Aliphatic | - | 0.22 | - | - |
| 64 | Heneicosane | Aliphatic | - | - | - | 1.93 |
| 65 | 1-Nonadecene | Aliphatic | - | - | - | 0.88 |
| 66 | n-Hexadecanoic acid | Aliphatic | - | 0.48 | - | - |
| 67 | Heneicosane | Aliphatic | - | - | 1.05 | - |
| 68 | 1-Nonadecene | Aliphatic | - | - | 0.58 | - |
| 69 | n-Hexadecanoic acid | Aliphatic | - | - | 0.91 | 0.54 |
| 70 | Heneicosane | Aliphatic | 0.98 | 0.65 | 0.91 | 1.54 |
| 71 | 1-Nonadecene | Aliphatic | - | - | 0.93 | 1.24 |
| 72 | 2-Nonadecanone | Aliphatic | - | - | 0.64 | 1.09 |
| 73 | Heneicosane | Aliphatic | 1.85 | 1.70 | 1.04 | 1.93 |
| 74 | 1-Nonadecene | Aliphatic | - | - | 0.56 | 0.59 |
| 75 | Heneicosane | Aliphatic | 3.54 | 3.76 | 0.87 | 1.89 |
| 76 | Octacosanol | Aliphatic | - | - | 0.61 | 1.24 |
| 77 | Behenic alcohol | Aliphatic | - | - | - | 0.29 |
| 78 | 2-Heptadecanone | Aliphatic | - | 0.14 | 0.73 | 1.50 |
| 79 | Octacosane | Aliphatic | - | 0.22 | - | - |
| 80 | Heneicosane | Aliphatic | 5.61 | 6.29 | 1.14 | 2.47 |
| 81 | Octacosanol | Aliphatic | - | - | 0.34 | 0.66 |
| 82 | Octacosane | Aliphatic | 0.34 | 0.58 | - | - |
| 83 | Octacosane | Aliphatic | - | 0.30 | - | - |
| 84 | Heneicosane | Aliphatic | 7.66 | 8.94 | 0.8 | 2.56 |
| 85 | Octacosanol | Aliphatic | - | - | - | 0.66 |
| 86 | 2-Heptadecanone | Aliphatic | - | - | - | 0.79 |
| 87 | Octacosane | Aliphatic | 0.48 | 1.03 | - | - |
| 88 | Pentacosane | Aliphatic | 0.50 | 0.72 | - | - |
| 89 | Heneicosane | Aliphatic | 8.56 | 10.18 | 1.06 | 3.24 |
| 90 | Behenic alcohol | Aliphatic | - | 0.16 | - | - |
| 91 | Octacosanol | Aliphatic | - | - | - | 0.49 |
| 92 | Tetracontane | Aliphatic | 0.50 | 1.54 | - | - |
| 93 | Octadecane, 3-methyl- | Aliphatic | - | 1.15 | - | - |
| 94 | Octacosane | Aliphatic | 0.69 | - | - | - |
| 95 | Heneicosane | Aliphatic | 9.90 | 11.70 | 0.88 | 3.19 |
| 96 | Behenic alcohol | Aliphatic | - | 0.32 | - | - |
| 97 | Tetracosanoic acid, methyl ester | Aliphatic | - | - | - | 2.21 |
| 98 | 2-Heptadecanone | Aliphatic | - | - | 0.48 | - |
| 99 | Tetracontane | Aliphatic | 0.40 | 0.96 | - | - |
| 100 | Octadecane, 3-methyl- | Aliphatic | - | 1.26 | - | - |
| 101 | Octacosane | Aliphatic | 0.85 | 0.31 | - | - |
| 102 | Squalene | Aliphatic | - | - | - | 1.09 |
| 103 | Heneicosane | Aliphatic | 10.23 | 11.76 | 0.82 | 3 |
| 104 | Octacosanol | Aliphatic | - | 0.41 | - | - |
| 105 | Octacosane | Aliphatic | - | 0.98 | - | - |
| 106 | Octadecane, 3-methyl- | Aliphatic | - | 1.1 | - | - |
| 107 | Tetracontane | Aliphatic | 9.56 | 11.16 | - | 1.86 |
| 108 | Octacosanol | Aliphatic | - | 0.34 | - | - |
| 109 | Hexacosanoic acid, methyl ester | Aliphatic | - | - | - | 2.63 |
| 110 | Tetracontane | Aliphatic | - | 0.36 | - | - |
| 111 | Nonacosane | Aliphatic | - | 0.71 | - | - |
| 112 | Tetracontane | Aliphatic | 7.28 | 8.09 | - | - |
| 113 | Octacosanol | Aliphatic | - | 0.35 | - | - |
| 114 | Tetracontane | Aliphatic | 2.14 | 3.25 | - | - |
| 115 | Tetracontane | Aliphatic | - | 1.24 | - | - |
| 116 | Tetracontane | Aliphatic | - | 1.95 | - | - |

The percentages of the unassigned compounds in rAHA, AHA-Cr(VI), PHA, and PHA-Cr(VI) are 8.92%, 4.03%, 24.27%, and 20.23% respectively.

**

**

**Figure S1.** Base titration curves for rAHA and PHA in 0.5N NaCl


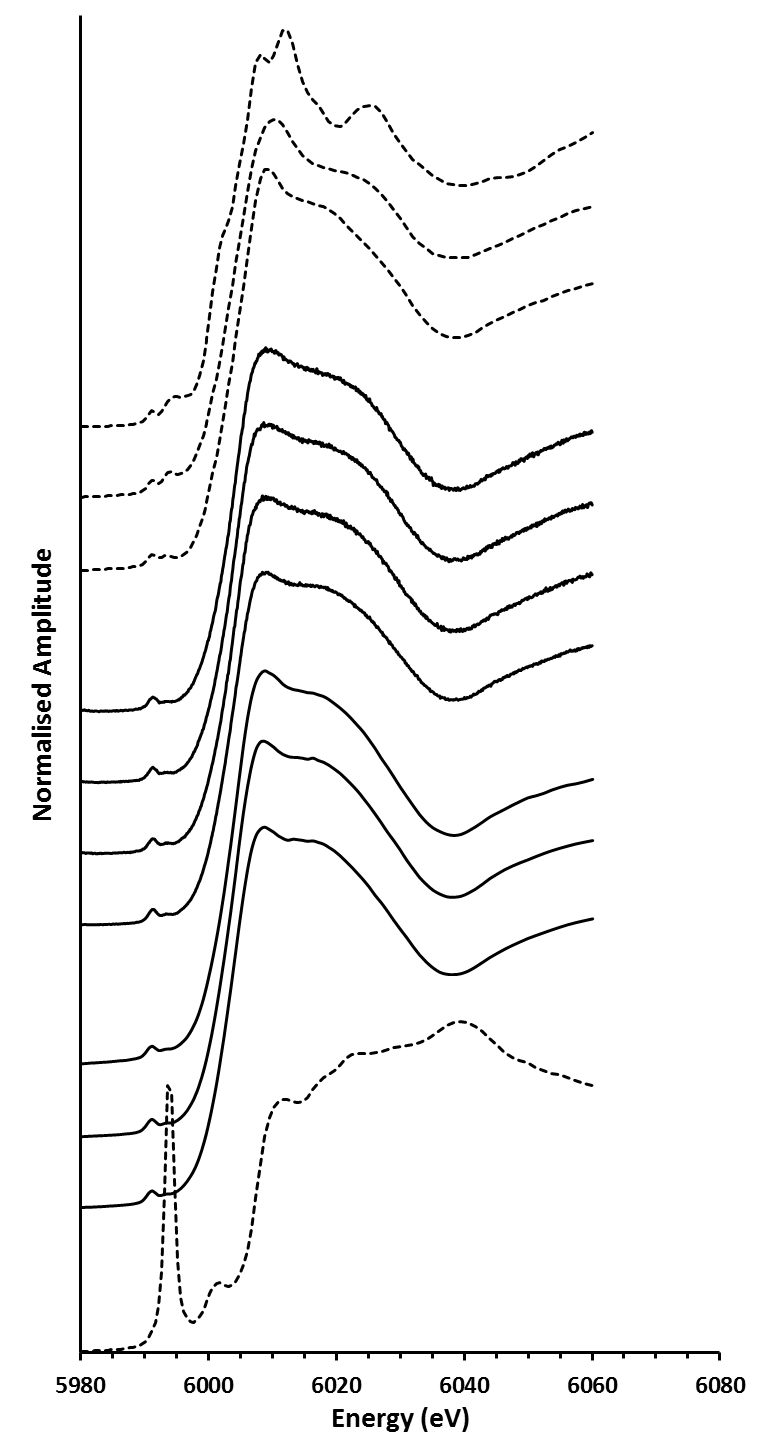


**AHA**

**PHA**

pH 7.6

pH 8.8

pH 10.5

pH 6.2

pH 7.8

pH 4.1

pH 3.7

Cr(OH)_3_

Cr_2_O_3_

Cr^3+^_(aq)_

CrO_4_^2-^_(aq)_

**Figure S2**. Cr K-edge XANES spectra collected from Aldrich (AHA) and peat (PHA) humic acid samples after reaction with chromate solution for 50 days, and from selected Cr(VI) and Cr(III) containing standards.

**Figure S3.** Pyrograms of rAHA at pH 3, AHA reacted with excess Cr(VI) initially at pH 3, PHA at pH 3 and PHA reacted excess Cr(VI) initially at pH 3. Peak identities and intensities are given in Table S7.

**Figure S4.** Pseudo first order rate constants and half-lives for the reduction of Cr(VI) by (a) AHA, and (b) PHA. Reactions contained 110 μmol Cr(VI)/g of HA, and an activity coefficient of unity has been assumed to calculate [H^+^] from the pH value. Dashed line indicates the pH value below and above which HCrO_4_^‑^ and CrO_4_^2‑^ are the dominant aqueous Cr(VI) species, respectively (Pourbaix, 1966).

**References**

Chen, Y., Senesi, N., Schnitzer, M., 1977. Information provided on humic substances by E4/E6 ratios. Soil Science Society of America Journal 41, 352-358.

Golchin, A., Clarke, P., Baldock, J., Higashi, T., Skjemstad, J., Oades, J., 1997. The effects of vegetation and burning on the chemical composition of soil organic matter in a volcanic ash soil as shown by 13C NMR spectroscopy. I. Whole soil and humic acid fraction. Geoderma 76, 155-174.

Janoš, P., Kříženecká, S., Madronová, L., 2008. Acid–base titration curves of solid humic acids. Reactive and Functional Polymers 68, 242-247.

Knicker, H., Totsche, K.U., Almendros, G., Gonzalez-Vila, F.J., 2005. Condensation degree of burnt peat and plant residues and the reliability of solid-state VACP MAS C-13 NMR spectra obtained from pyrogenic humic material. Organic Geochemistry 36, 1359-1377.

Kögel-Knabner, I., 2000. Analytical approaches for characterizing soil organic matter. Organic Geochemistry 31, 609-625.

Pourbaix, M., 1966. Atlas of electrochemical equilibria in aqueous solutions. Pergamon Press, Oxford

Ravel, B., Newville, M., 2005. ATHENA, ARTEMIS, HEPHAESTUS: data analysis for X-ray absorption spectroscopy using IFEFFIT. Journal of synchrotron radiation 12, 537-541.

Ritchie, J.D., Perdue, E.M., 2003. Proton-binding study of standard and reference fulvic acids, humic acids, and natural organic matter. Geochimica et Cosmochimica Acta 67, 85-96.

USEPA, 1992. SW-846 Manual: Method 7196A. Chromium hexavalent (colorimetric).

USEPA, 1996. Method 3050B: Acid Digestion of Sediments Sludges and Soils (revision 2).
